# Enabling technologies driving drug research and development

**DOI:** 10.3389/fmed.2023.1122405

**Published:** 2023-03-09

**Authors:** Panna Vass, Dara Sevkan Akdag, Gabriel Enemark Broholm, Jesper Kjaer, Anthony J. Humphreys, Falk Ehmann

**Affiliations:** ^1^Regulatory Science and Innovation Task Force, European Medicines Agency, Amsterdam, Netherlands; ^2^Data Analytics Centre, Danish Medicines Agency, Copenhagen, Denmark

**Keywords:** enabling technologies, regulatory science, European Medicines Agency, Innovation Task Force, European Medicines Regulatory Network

## Abstract

One of the strategic goals of the European Medicines Agency (EMA) and the European Medicines Regulatory Network is to support the research and uptake of innovative methods and technologies in the development of medicines. To promote this goal, EMA drew up a list of enabling technologies (ETs), which are novel and fast-growing technologies that have the potential to enable innovation and therefore exert considerable impact on drug development. In this work, enabling technologies identified by the EMA are analysed to measure their impact on drug development by following their journey from publications through early regulatory interactions to clinical trials between 2019 and 2022. This work also reviews the current list of EMA-identified ETs by scrutinising previously unseen innovative technologies identified in EMA submissions data. The analysis shows large variations in the appearance of the various innovative technologies in the different studied data sources, which provided valuable insights into the “Journey of Innovation” that innovative technologies undergo. Several emerging technologies were identified and endorsed for inclusion in the enabling technologies list, whereas some others already on the list were proposed to be excluded due to their low appearance in regulatory interactions as well as clinical trials and publications. Overall, this analysis highlights the relevance and value of continuously scanning and monitoring enabling technologies, supporting Europe’s goal to remain a leader in research and development of innovative technologies, methods, and methodologies relevant to drug development.

## Introduction

One of the strategic goals of the European Medicines Agency (EMA) and the European Medicines Regulatory Network is to foster research and the uptake of innovative methods in the development of medicines, as set out in the “Regulatory Science Strategy to 2025” (1) and the “European Medicines Agencies Network Strategy to 2025” (2). Supporting this goal, assistance and guidance are provided across the EU regulatory network through various initiatives and procedures, including Scientific Advice, Innovation Office meetings, Qualification of Novel Methodologies, and specific support for Small and Medium Sized Enterprises (SMEs). The EMA’s Innovation Task Force (ITF) (3) offers an early contact point for developers for an informal dialogue on innovative scientific, technical or legal aspects in medicines development. These early interactions enable the EMA to get an insight into emerging enabling technologies (ETs). This is also supported by Horizon Scanning initiatives at the Agency and other organisations (Supplementary Table S1) that systematically examine information from various sources to detect early signs of important and potentially disruptive developments in the public health space (4, 5).

ETs are defined as novel and fast-growing technologies that have the potential to enable innovation and therefore exert considerable impact on drug development (6, 7). ETs have a broad range of applications across multiple industries and fields, allowing for the creation of new, more efficient solutions and platforms to tackle societal challenges. ETs are highly adaptable and can often be combined with other technologies to create even more powerful solutions.

An example of ETs is artificial intelligence (AI), which, in the context of health, can be used to analyse large amounts of data and identify relevant patterns efficiently. AI tools can be utilised in many ways in drug research, discovery and development (8, 9). For instance, AI can make the discovery process faster, more efficient and cost-effective, better targeted, and more specific, e.g., by utilising computational drug screening (10–12). Another example of ETs is genetic modification or genome editing, which have the potential to transform how certain diseases are treated or potentially cured. Therapies based on these ETs (e.g., cell and gene therapies) can stop or slow down the effects of diseases by targeting them, e.g., at the genetic level (13). Additionally, molecular matching of patients to treatments is also possible, given that the genetic driver for the disease is known (10). Another ET, advanced manufacturing, including continuous manufacturing or 3D printing, could be essential in public health emergency preparedness and response. Innovations in manufacturing technologies can lead to integrated processes with fewer steps, consistency and shorter processing times. They can also support enhanced development methodologies that enable real-time product quality monitoring, e.g., by quality by design (QbD) approaches and the use of process analytical technology (PAT) tools and models. Advanced manufacturing technologies also enable flexible processes that allow scale-up, scale-down, and scale-out to accommodate varying demands in supply (14).

The European Medicines Agency drew up a list of ETs (Supplementary Table S2) in 2016, based on knowledge at that time. A cross-Agency multidisciplinary team analysed the previous submissions and stakeholder interactions and agreed to the list of ETs. Since then, ITF and other EMA procedures, including Scientific Advice (15), Orphan applications (16) and Qualification of novel methodologies (17), have been collecting data on ETs.

The tracking of ETs in regulatory interactions, clinical trials and publications, and the exploration of the collected data is highly valuable as it supports the continuous future-proofing of the Agency. By analysing the information on ETs, science and technology trends in innovative drug development can be detected early on, allowing the Agency to focus on relevant, most promising developments.

Given its relevance, it is critical that the list of ETs is up-to-date. The field of drug development is rapidly changing, and the emergence of novel ETs is continuously ongoing. Additionally, the development and adoption of novel technologies in the pharmaceutical industry in Europe are progressing at different speeds across the various stakeholders in the field (large pharma, SMEs, academia, etc.) driven by a whole set of changing priorities, challenges, and use cases. For instance, this process has been impacted by the COVID-19 pandemic, resulting in the expedited development and adoption of some innovative technologies, like mRNA-based vaccines and digital technologies (18). These factors justify the investment for an up-to-date list of ETs to incorporate novel and evolving ETs and remove ETs that prove to be less impactful than previously expected.

The aim of this work was two-fold: on the one hand, it was aimed to measure the impact of the identified ETs by following and analysing their route (i.e., the “Journey of Innovation”) from publications through early interactions with EMA to clinical trials (CTs). On the other hand, this work also aimed to review and suggest an update to the current list of ETs with previously unseen innovative technologies identified during the data analysis to further strengthen the support of EMA for the development of innovative technologies, methods, and methodologies.

## Methods

### Search and analysis of publications

Scientific publications related to the various ETs were searched in the PubMed databases first without any restrictions, using different search terms for each ET. As these searches resulted in a very large number of hits (generally >10.000), the results were limited by using the PubMed Advanced Search Builder applying the following methods:

Searches were restricted to publications with a publication date between 2019 and 2022.
Searches were restricted to publications in English language.
Searches were narrowed down by using a combination of more specific search terms (limiting them to titles and abstracts) and MeSH terms or Major MeSH terms.

The aim was to try to capture the widest possible range of publications related to a specific ET, including basic science publications, as eventually the results basic science might also be used in drug development.

The accuracy of the searches was evaluated by screening the first approx. 20–50 hits received after a search. The search terms were attempted to be modified in a way that

The search terms would be broad enough to minimise the risk of losing relevant publications
The results would include only a small percentage of irrelevant publications.

The list of search terms and the number of hits can be found in Supplementary Table S3. The ETs were regrouped compared to the original ET list in a way that the new groups are more descriptive of the ETs within the group. and these groups were used in the Results section. The grouping can be found in Supplementary Table S5.

In one case (ET: “Mobile/portable manufacturing”), no hits were returned after the search despite using various search terms. To validate this, a more generic search engine (Google) was also used.

### Analysis of EMA data

Requests with associated ETs received by the various EMA procedures [ITF and other EMA procedures (Scientific Advice, Protocol Assistance, Qualification of Novel Methodologies, Orphan Designation)] between 2019 and 2022 were collected from EMA databases (ITF and IRIS databases). The ITF database collects the information submitted in the “ITF meeting request form” by the applicants to the Agency (19). IRIS is EMA’s online regulatory and scientific information management platform, through which certain regulatory procedures (e.g., Scientific Advice, Orphan Designation) are carried out (20).

In the ITF database, the enabling technologies selected in the application form for each ITF request are recorded. In the IRIS database, Research Product Identifiers (RPI) are associated to enabling technologies by referential terms associations. In both databases, products/developments can have multiple ETs associated to them. The ITF requests and RPIs associated to certain enabling technologies were listed and the occurrence of each enabling technology was counted. Applications without any selected ETs or with “Other innovation aspect / enabling technology” selected were collected and inspected individually to identify the associated –potentially novel –ETs.

### Clinical trials search

All clinical trial searches were carried out in the ClinicalTrials.gov database using the expert search function. Post-marketing surveillance trials (Phase 4) were excluded from the searches. The first 20–30 search results were inspected, and the search terms were modified similarly to the PubMed searches to maximise search accuracy.

The list of search terms and the number of hits can be found in Supplementary Table S4. The ETs were regrouped compared to the original ET list for clarity and these groups were used in the Results section. The grouping can be found in Supplementary Table S5.

The main aim of the ClinicalTrials.gov database is to report summary information about clinical study protocols and results. The database was not set up to record information related to technologies and this might lead to underreporting of enabling technology use in clinical trials.

## Results

### Publications

Table 1 contains the list of the ETs along with the number of associated publications with them based on the PubMed searches. Two ETs, E/m health (Digital healthcare) and Novel biomarkers/omics were linked to a particularly high number of publications (more than 20,000 and 10,000, respectively), despite the fact that the search was limited by using the advanced search function and optimised search terms. Other ETs with a high number of publications included Novel data sources, Associated medical devices, and Genome editing.

**Table 1:** Enabling technologies in PubMed.

Enabling technologies	Search hits
Digital healthcare	24,479
Novel biomarkers, omics	10,480
Novel data sources	7,867
Associated medical devices	6,658
Genome editing	3,523
Smart materials and active substance(s)	2,839
Nanotechnologies	2,812
Novel non-clinical development methods	2,760
Other ingredients	1992
Delivery methods	1802
Biomaterials	1721
Advanced manufacturing	1,261
Novel clinical trial methodologies	1,068
Synthetic biology	712
Genetically modified organism(s)	441
Development-related: clinical	305
Transgenic technologies	216
Human cell-based *in vitro* models	51

List of enabling technology categories with the number of associated publications found in PubMed searches using optimised search terms. Publications were retrieved by searching in the PubMed database using search strings described in Supplementary Table 3 and limiting the results to English publications between 2019 and 2022. The coloring of the cells is based on the value in the given cell. Lighter means lower value and darker means higher value.

Interestingly, search for Mobile/portable manufacturing resulted in 0 hits. To “validate” this result, an additional Google search was carried out. The first 10 results in Google concerned a portable pharmaceutical manufacturing system development at MIT in 2016 and the Portable Continuous Miniature Modular (PCMM) manufacturing platform developed by Pfizer in 2015. Only one of the top 20 results was more recent than these: a small-scale rapid mobile manufacturing platform by Moderna from 2020 (21).

### EMA databases

Table 2 contains the ET categories along with their relative frequencies in the EMA datasets. The most often raised topics by ITF applicants during the analysed 3 years were associated with novel clinical trial methodologies, digital healthcare, delivery methods, novel biomarkers, omics, and advanced manufacturing. Fairly similar trends were observed in the other EMA datasets, some of the most often referenced ET categories being novel clinical trial methodologies, delivery methods, and advanced manufacturing. For both ITF and other EMA applications, “Other innovation aspect/enabling technology” was amongst the most frequently chosen categories justifying a further investigation into the specific ETs behind this category.

**Table 2:** Enabling technologies in EMA databases.

Enabling technologies	ITF	Other EMA procedures
Novel clinical trial methodologies	11%	12%
Other innovation aspect / enabling technology	10%	11%
Digital healthcare	9%	4%
Delivery methods	9%	11%
Novel biomarkers, omics	8%	3%
Advanced manufacturing	6%	7%
Genome editing	6%	6%
Associated medical devices	6%	3%
Novel non-clinical development methods	5%	4%
Novel data sources	5%	3%
Genetically modified organism(s)	5%	13%
Nanotechnologies	5%	3%
Biomaterials	4%	2%
Synthetic biology	3%	3%
Transgenic technologies	3%	6%
Human cell-based *in vitro* models	2%	4%
Other ingredients	2%	4%
Smart materials and active substance(s)	1%	1%
Development-related: clinical	0%	1%

Relative frequencies of enabling technology categories in ITF and other EMA requests received between 2019 and 2022. The coloring of the cells is based on the value in the given cell. Lighter means lower value and darker means higher value.

Biodefense/biowarfare, Medicines for tropical diseases, Other smart/advanced material, and Photodynamic product were found to be the least often selected ETs by developers in both ITF and other EMA applications.

Some differences were also observed in the data: only 5% of the ITF applications were related to genetically modified organism(s), whereas it was the most frequently referenced category (13% of the requests) in the other EMA datasets. Moreover, digital healthcare and novel biomarkers, omics were amongst the most selected categories (around 8–9%) amongst the ITF applicants. In contrast, only 3–4% of the applicants to other EMA procedures referenced the same categories.

#### Other innovation aspect/enabling technology category

As it can be seen in Table 2, the Other enabling technologies category was amongst the most selected ETs in the EMA databases. This category was analysed in more detail as this is the category that applicants choose if their development does not fit any of the other categories and therefore could contain previously unidentified innovation. Requests without any selected ETs or with “Other innovation aspect/enabling technology” selected were collected and inspected individually to manually identify associated innovative topics. 10% of these requests concerned medical devices or *in-vitro* diagnostics. Other notable themes identified in these 39 requests were artificial intelligence/machine learning, platform technologies, faecal microbiota transplantation (FMT), exosomes, and developments related to special populations, amongst others.

### Clinical trials

The table below (Table 3) gives information about the number of clinical trials associated with the various ETs. The Associated medical devices category had the highest number of related clinical trials, with considerably more hits (>3,000) than the categories following it (Matrices and Adjuvant (Other ingredients), Nanotechnologies and Digital healthcare) (450–800 hits).

**Table 3:** Enabling technologies in clinical trials.

Enabling technologies	Search hits
Associated medical devices	3,825
Other ingredients	819
Nanotechnologies	533
Digital healthcare	456
Novel clinical trial methodologies	391
Smart materials and active substance(s)	356
Delivery methods	292
Novel data sources	135
Novel biomarkers, omics	72
Biomaterials	53
Transgenic technologies	53
Novel non-clinical development methods	43
Development-related: clinical	38
Advanced manufacturing	9
Genome editing	8
Synthetic biology	4
Genetically modified organism(s)	2
Human cell-based *in vitro* models	1

List of enabling technologies with the number of clinical trials associated with them. Trials were retrieved by searching all interventional trials (post-marketing surveillance trials were excluded) in the clinicaltrials.gov database using optimised search terms containing the various enabling technologies described in Supplementary Table 4. The coloring of the cells is based on the value in the given cell. Lighter means lower value and darker means higher value.

It is to be noted that no clinical trials were found to be associated with manufacturing-related ETs (Bedside/point of care manufacturing, Distributed manufacturing, Portable manufacturing), except for 3D printing, which had 9 associated clinical trials.

### Comparison of the data sources

**Figure 1:**
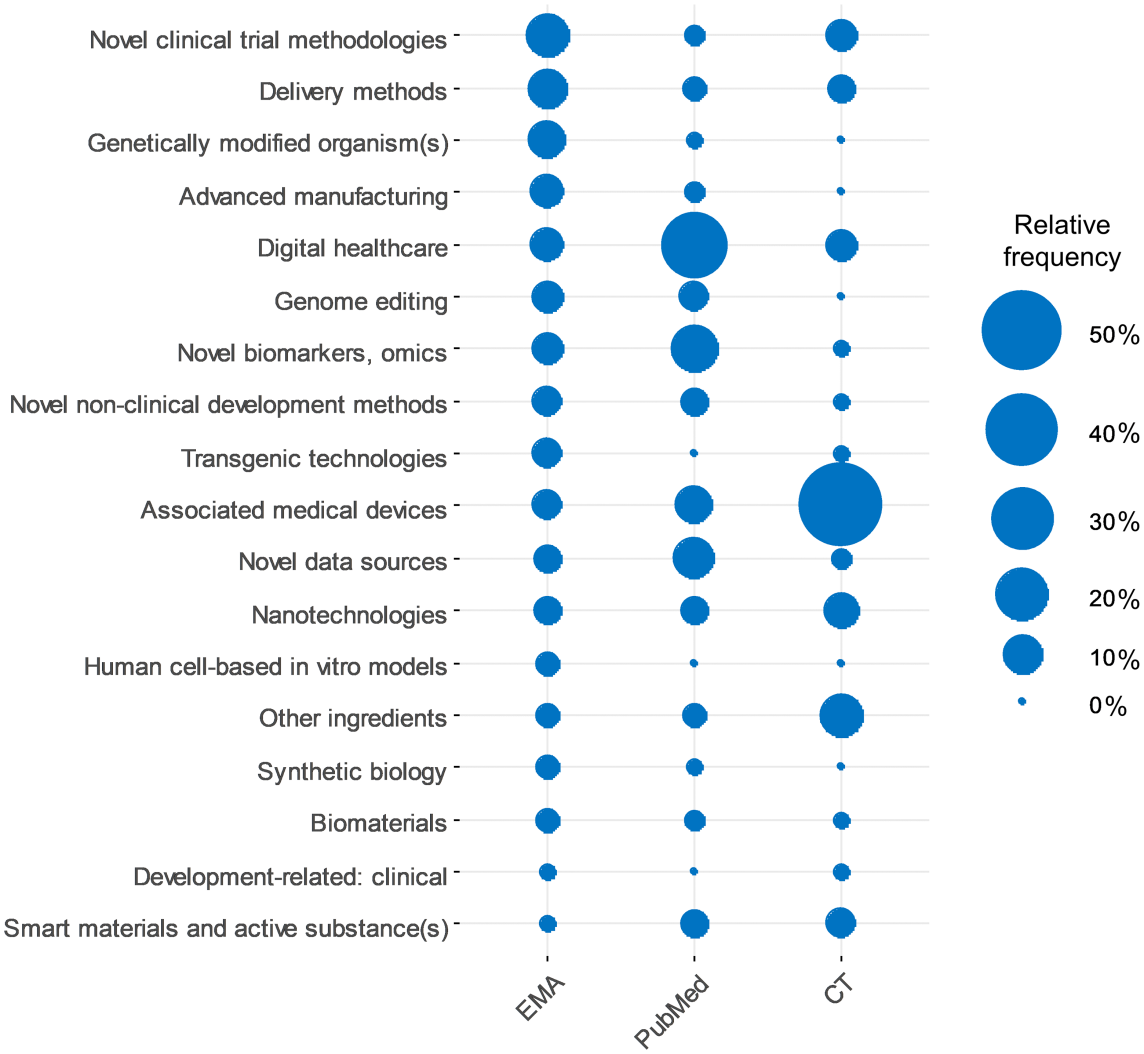
Comparison of enabling technologies in different data sources. Relative frequencies of enabling technology categories in EMA requests, PubMed and clinical trials searches. The category of “Other innovation aspect/enabling technology” was omitted as it is not applicable in the case of publications and clinical trials.

Figure 1 shows the ET categories along with their relative frequencies in the various data sources analysed in this work (EMA databases, PubMed search, and clinical trial database results). There are differences between the different data sources in terms of the most and least frequently referenced ETs.

Several ETs had very low appearances across the different data sources. Such ETs included biodefense/biowarfare, Avatar, nude and humanised mice, Bioenhancer, Pharmacological chaperone, Photodynamic product, Printing, and Mobile/portable manufacturing.

## Discussion

The European Pharma Strategy highlights that in order to guarantee that high-quality and safe medicines reach patients it is crucial that the European regulatory environment understands and facilitates innovation (22). Support for innovation is an important goal at EMA and the European Medicines Regulatory Network (EMRN), as highlighted in the EMA’s Regulatory Science Strategy to 2025 (1) and the European Medicines Agencies Network Strategy (2). Identification, monitoring and continuous updating of emerging innovative technologies (ETs), methods, and methodologies enables their use in innovative drug development. The identification of trends further supports the allocation of resources and specialised expertise within the network.

The EMA list of identified enabling technologies has been used by the Innovation Task Force for more than 5 years and more recently, by other EMA procedures, including Scientific Advice, Orphan Designation, Qualification of Novel Methodologies, and Business Pipeline Meetings. In this work, data accumulated over the past 3 years on ETs identified by the EMA was analysed to measure their impact on drug development by following their journey from publications through early regulatory interactions to clinical trials. This study also reviewed the current list of ETs by scrutinising previously unseen innovative technologies identified in EMA submissions data.

We identified differences when analysing the appearance of ETs in different data sources over the studied time period (Figure 1). A reason for this could be that innovative developments are likely to appear in the various analysed data sources (publications (PubMed), early regulatory interactions, e.g., EMA ITF, and clinical trials) at different times based on their development stage/maturity, which reflects the “Journey of Innovation” that these ETs undergo. The majority of the ET categories were well represented in at least one data source, which confirms these technologies’ relevance. However, several ETs had very low appearance across the different data sources, which could indicate that either even though they showed potential for growth earlier, later their development was slowed down/abandoned or that the development of these specific ETs has not been realised yet. The need for continued availability of these terms should be reassessed. Attention should be paid to the “Other innovation aspect / enabling technology” section to be able to detect if the development of any of the ETs is enhanced. The list of ETs should be reviewed periodically to cheque the relevance of ETs. Given the extent of data being produced in the field of drug development (publications, regulatory submissions or clinical trials), quantitative/automatic approaches, like machine learning or natural language processing, should be considered to summarise/track developments of ETs.

Digital healthcare has been frequently identified in all studied data sources, including EMA procedures, publications and clinical trials (Figure 1). There is ongoing global growth in digitalisation in all sectors (23). The European Commission’s Competence Centre on Foresight identified digitalisation in healthcare as part of a Megatrend, defined as “long-term driving forces that are observable now and will most likely have a global impact” (24). Recognising this, the EMA and the EU Regulatory Network provide specific support in this area (25–27). EMA has issued several qualification opinions and advice for digital technology-based methodologies as part of the Qualification of novel methodologies for medicine development (QoNM) pathway, which is intended to support the qualification of innovative development methods for a specific intended use in the context of research and development into pharmaceuticals (28, 29). Qualified digital technologies, amongst others, include an ingestible sensor that can monitor medication adherence of patients, a system that allows the capture of clinical study source data electronically, and a registry for pharmacoepidemiology studies (30). To further support the successful development of digital technology-based methodologies, EMA has released a question and answer document focusing on the qualification of digital technologies (31) and has started a focus group on the QoNM framework and an AI coordination group. During the COVID-19 pandemic, many digital health tools moved from being seen as a potential opportunity to an immediate need, and their use increased considerably (32). Other activities and developments are expected in this field if the current momentum is retained as the pandemic comes under control, and therefore the ETs related to Digital healthcare (e.g., E/m-health and Monitoring devices/sensors/systems) could be reviewed and subdivided into more granular terms.

The lack of clinical trials associated with manufacturing-related ETs could be due to the fact that manufacturing-related ETs are not relevant for clinical trials. In fact, an ET is an attribute of a product, not necessarily of a clinical trial or an EMA submission. An ET associated to a product may be relevant for some but not all CTs carried out with the product.

The adoption of decentralised clinical trials has been increasing over the past few years, enabled further by the advancement of available technologies. The relatively high number of medical devices (MD) and *in-vitro* diagnostics (IVD)-related requests could be linked to the use of sensors like glucometers or wearable activity trackers or eDiaries, which are decentralised clinical trial enablers. In addition, there are two new EU legislations in this area (MDR (33) and IVDR (34)), which entered into force in 2021 and 2022. The new Regulations introduced new tasks and responsibilities for EMA, for example in the assessment of combination products and companion diagnostics, in the monitoring of medical device shortages, and in the support of the various medical device expert panels. These tasks require strong liaison between EMA, EU member states, and notified bodies, which indicates the need for the two regulatory frameworks (i.e., notified bodies and medicines regulators) to collaborate and work well together. Based on the increasing exposure, the number of MD and IVD-related requests at the EMA is expected to grow further. The Associated medical devices category in the ET list covers Matrices, Other associated medical device, and Biomaterials; however, these might not cover all aspects of MDs and IVDs. Therefore, it is recommended to either refine the already existing ETs or to include a separate MD/IVD-related ET in the list.

Analysis of EMA procedures without ETs flagged or the category “Other innovation aspect / enabling technology” offered insights into what terms or categories might be missing from the current ETs list. Newly identified technologies/methods/methodologies and treatment mondalities included artificial intelligence and machine learning, manufacturing platform technologies, faecal microbiota transplantation (FMT), developments related to special populations, e.g., pregnant women, a novel environmental monitoring technology, and exosomes. Several of these technologies are described below. It should be noted that applicants may not always choose sufficient or correct ETs when submitting applications, which could be mitigated by adding more detailed instructions to the application forms to help applicants choose the most suitable ET.

Artificial Intelligence and Machine Learning were amongst the new ETs, and their appearance is in line with the general trend of increasing application of AI technologies in medicines development (4). As these technologies are missing from the ET list –the closest related term is Big data analysis –it is suggested to include them in the list given their significant potential for growth and impact in the upcoming years (35).

A notable theme identified in the uncategorised requests was related to platform technologies. One of the main characteristics of platform technologies is flexibility, which makes them well suited to fight against novel pathogens or diseases that are sporadic or unpredictable. In 2016, following a public consultation, the World Health Organisation (WHO) presented six promising platforms to increase R&D preparedness for future epidemics (36). It is expected that future epidemics will become more frequent, more complex and challenging to prevent and contain, and therefore the efforts to develop platform technologies are also likely to be increased in the future (12, 37).

FMT was also mentioned in the “Other innovation aspect / enabling technology” category. FMT has gained considerable interest in the past decades due to the high recovery rate in recurrent *Clostridium difficile* infections compared to traditional antibiotic therapy. Research and development of FMT-based products have advanced in recent years worldwide. A recent EU-Innovation Network Horizon Scanning report on FMT anticipates further developments in the field, with potential Marketing Authorisation Applications happening in the EU within the next 5–10 years (38).

Exosomes and extracellular vesicles were another novel technology category identified. Extracellular vesicles have recently attracted great attention as they act as intercellular communication signalling mediators. They can also carry a wide range of cargo molecules and have the potential to act as diagnostic markers in clinical applications. Despite the growing interest in exosome or extracellular vesicle-based therapies, the technologies are still in their infancy, and regulatory requirements for these products need to be clarified by regulators worldwide. The Pharmaceuticals and Medical Devices Agency (PMDA) in Japan, in collaboration with the International Coalition of Medicines Regulatory Authorities (ICMRA), is developing a “regulatory points to consider” document to address the open questions in the field (39).

Except for medical devices/*in-vitro* diagnostics and artificial intelligence/machine learning, which were associated with a large number of requests, the other technologies (platform technologies, FMT, and exosomes) appeared in a small number of EMA submissions (generally 1–3 request(s)/new ET). Therefore, these terms could be considered weak signals and have to be tracked to evaluate if a new ET should be added to the list.

In summary, this work explored the presence of ETs in EMA submissions, analysing their relevance with respect to publications and clinical trials. This provided valuable insight into the “Journey of Innovation and Trends” from publications over early interactions with the Regulator to clinical trials. This work also reviewed the current list of EMA ETs and by scrutinising EMA data submissions, previously unseen innovative technologies were identified, resulting in the following recommendations:

Analysis of “Other innovation aspect / enabling technology” category in ITF resulted in suggestions for new ET terms, e.g., MDs/IVDs, artificial intelligence/machine learning. Potential future ETs could include platform technologies, FMT, and exosomes.
The enabling technologies related to Digital healthcare (e.g., E/m-health and Monitoring devices/sensors/systems) could be reviewed and subdivided into more granular terms.
Some of the enabling technologies had very low appearances across the studied data sources; therefore, the need for their continued availability in the list should be reassessed (e.g., Biodefense/biowarfare, Pharmacological chaperone).
A mechanism for systematically updating the ETs list in a quantitative way [e.g., with natural language processing (NLP)].

The design of the current analysis is subject to limitations that could be addressed in future research. This cross-sectional study of ETs in EMA submissions, publications and clinical trials looked at a 3-year long time period. A longitudinal review could be carried out, including the interpretation of the time between the identification of the same ET in the different sources, which would allow for the charting of the journey of certain ETs from basic science to their use in regulatory contexts. A more progressive/sensitive methodology could also be considered involving the analysis of patents, venture capital investments, and more dynamic data sources (e.g., Google, Wikipedia), and social media (e.g., Twitter, Reddit). Additionally, the examination of the historical events, policies, and regulations that have shaped the development of an ET can provide a deeper understanding of its evolution. The EMA and the members of the EMRN are digital-driven medicines regulators that continuously take advantage of innovations and emerging technologies for the ultimate benefit of public and animal health, and therefore the exploration of machine learning methodologies, e.g., text mining algorithms for the identification of novel ETs in regulatory and public data would also be of high interest.

## Data availability statement

The original contributions presented in the study are included in the article/Supplementary material, further inquiries can be directed to the corresponding authors.

## Author contributions

PV, FE, and AH contributed to the conception and design of the study. PV performed the research and analysed the data. PV and FE wrote the first draft of the manuscript. All authors contributed to the critical revision of the manuscript, read, and approved the submitted version.

## Conflict of interest

The authors declare that the research was conducted in the absence of any commercial or financial relationships that could be construed as a potential conflict of interest.

## Publisher’s note

All claims expressed in this article are solely those of the authors and do not necessarily represent those of their affiliated organizations, or those of the publisher, the editors and the reviewers. Any product that may be evaluated in this article, or claim that may be made by its manufacturer, is not guaranteed or endorsed by the publisher.
