# Supplementary material for: Enabling technologies driving drug research and development
Source: Front Med (Lausanne). 2023 Mar 9;10:1122405. doi: 10.3389/fmed.2023.1122405 (PMC10033683; doi:10.3389/fmed.2023.1122405)
Supplement: Supplementary file 1 [file Table_1.docx]

Enabling technologies driving drug research and development.

**Panna Vass^1*^, Dara Akdag^2^, Gabriel Enemark Broholm^2^, Jesper Kjaer^2^, Anthony J. Humphreys^1^, Falk Ehmann^1*^**

1, Regulatory Science and Innovation Task Force, European Medicines Agency

2, Data Analytics Centre, Danish Medicines Agency

# Supplementary material

Supplementary Table 1. List of horizon scanning initiatives.

| Organisation | Link |
| --- | --- |
| European Medicines Agency (EMA) | [Horizon Scanning](https://www.ema.europa.eu/en/human-regulatory/research-development/innovation-medicines) |
| EU Innovation Network (EU-IN) | [Horizon Scanning](https://www.hma.eu/about-hma/working-groups/eu-innovation-network-eu-in.html) |
| European Commission (EC) | [Competence Centre on Foresight](https://knowledge4policy.ec.europa.eu/foresight/topic/horizon-scanning_en) |
| World Health Organisation (WHO) | [WHO Foresight](https://www.who.int/activities/monitoring-emerging-technologies-and-building-futures-thinking-who-foresight) |
| OECD Observatory of Public Sector Innovation | [Futures & Foresight](https://oecd-opsi.org/guide/futures-and-foresight/) |
| International Horizon Scanning Initiative (IHSI) | [Horizon Scanning System](https://ihsi-health.org/horizon-scanning-system/) |
| International Coalition of Medicines Regulatory Authorities (ICMRA) | [Informal Network for Innovation](https://www.icmra.info/drupal/en/strategicinitiatives/innovation) |
| European Network for Health Technology Assessment (EUnetHTA) | [Horizon Scanning](https://www.eunethta.eu/ja3services/horizon-scanning/) |

Supplementary Table 2. List of enabling technologies at the European Medicines Agency.

| Enabling technology categories | Enabling technologies |
| --- | --- |
| Advanced manufacturing | 3D printing |
|  | Bedside/point of care manufacturing |
|  | Distributed manufacturing |
|  | Mobile/portable manufacturing |
|  | Printing |
|  | Transgenic technologies |
| Associated medical devices | Matrixes |
|  | Other associated medical device |
|  | Biomaterials |
| Delivery methods | Controlled-release technologies |
|  | New/uncommon pharm. form or route of admin. |
|  | Targeted release to specific site(s) |
| Development-related: clinical | Biodefense/biowarfare |
|  | Medicines for tropical diseases |
|  | Novel biomarkers, omics |
| Digital healthcare | Closed loop systems |
|  | E/m-health |
|  | Monitoring devices/sensors/systems |
| Directly product related | Genetically modified organism(s) |
|  | Synthetic biology |
|  | Nanotechnologies |
| Genome editing | Genome editing - deletion |
|  | Genome editing - regulation |
|  | Genome editing - replacement |
| Human cell-based | Human cell based in vitro models |
|  | Human stem cell in vitro models |
| Methodology of clinical trials | Adaptive designs |
|  | Bayesian designs |
|  | Extrapolation proposed |
|  | Novel endpoints |
|  | Platform/Umbrella/basket trials |
| Non-clinical development: other | Avatar, nude and humanised mice |
|  | Organoids |
|  | Other in silico models |
|  | Physiologically-based pharmacokinetics |
| Novel data sources | Big data analysis |
|  | Real world data analysis |
| Other ingredients | Adjuvant |
|  | Bioenhancer |
|  | Novel/uncommon excipient |
|  | Pharmacological chaperone |
| Other innovation aspect / enabling technology | Other innovation aspect / enabling technology |
| Smart materials and active substance(s) | Other smart/advanced material |
|  | Photodynamic product |

Supplementary Table 3. PubMed search terms

|  | **Search hits** | **Search terms** |
| --- | --- | --- |
| E/m-health | 21491 | ((ehealth[Title/Abstract]) OR (e health[Title/Abstract]) OR (electronic health[Title/Abstract]) OR (mhealth[Title/Abstract]) OR (m health[Title/Abstract]) OR (mobile health[Title/Abstract])) AND ((english[Filter]) AND (2019:2022[pdat])) |
| Novel biomarkers, omics | 10480 | "Biomarkers"[Majr:noexp] AND ((english[Filter]) AND (2019:2022[pdat])) |
| Matrixes | 6401 | (matri*[Title/Abstract]) AND (("Pharmaceutical Preparations"[Mesh]) OR ("Drug Delivery Systems"[Mesh])) AND ((english[Filter]) AND (2019:2022[pdat])) |
| Real world data analysis | 4953 | (((real world data[Title/Abstract]) OR (rwe[Title/Abstract])) OR (real word evidence[Title/Abstract])) OR (rwd[Title/Abstract]) AND ((english[Filter]) AND (2019:2022[pdat])) |
| Genome editing (all subcategories) | 3523 | (("Gene Editing"[Mesh]) AND (genom*[Title/Abstract])) NOT ("Plants"[Mesh]) AND ((english[Filter]) AND (2019:2022[pdat])) |
| Big data analysis | 2914 | ((big data[Title/Abstract]) AND ("Humans"[Mesh])) AND ((english[Filter]) AND (2019:2022[pdat])) |
| Nanotechnologies | 2812 | (("Nanotechnology/methods"[Mesh] OR "Nanotechnology/trends"[Mesh])) AND ((english[Filter]) AND (2019:2022[pdat])) |
| Monitoring devices / sensors /systems | 2681 | (("Monitoring, Physiologic/instrumentation"[Mesh]) OR ("Monitoring, Physiologic/trends"[Mesh])) AND ((english[Filter]) AND (2019:2022[pdat])) |
| Photodynamic product | 2561 | "Photochemotherapy/methods"[Mesh] AND ((english[Filter]) AND (2019:2022[pdat])) |
| Organoids | 1757 | "Organoids"[Majr] AND ((english[Filter]) AND (2019:2022[pdat])) |
| Biomaterials | 1721 | (("Biocompatible Materials"[Majr]) AND (("Pharmaceutical Preparations"[Mesh]) OR ("Drug Delivery Systems"[Mesh]) OR ("Drug Development"[Mesh]) OR ("drug development"[Title/Abstract]) OR ("pharmaceutical development"[Title/Abstract])) AND ((english[Filter]) AND (2019:2022[pdat]))) |
| Targeted release to specific site(s) | 1311 | ((("targeted release"[Title/Abstract]) OR("targeted deliver*"[Title/Abstract])) AND ("Drug Delivery Systems"[Mesh])) AND ((english[Filter]) AND (2019:2022[pdat])) |
| Bioenhancer | 1236 | ((bioenhancer*[tiab]) OR (enhanc*[tiab]) OR (increas*[tiab])) AND (("Biological Availability"[Mesh]) AND (drug[tiab])) AND ((english[Filter]) AND (2019:2022[pdat])) NOT ("Dietary Supplements"[Mesh]) |
| 3D printing | 1192 | ((((3D[Title/Abstract]) OR (three-dimensional[Title/Abstract]) OR (3 dimensional[Title/Abstract])) AND (print*[Title/Abstract])) OR ("Printing, Three-Dimensional"[Majr])) AND (("Pharmaceutical Preparations"[Mesh]) OR ("Technology, Pharmaceutical"[Mesh]) OR ("Drug Development"[Mesh])) AND ((english[Filter]) AND (2019:2022[pdat])) |
| Synthetic biology | 712 | Synthetic Biology[Majr] NOT Plants[Mesh]AND ((english[Filter]) AND (2019:2022[pdat])) |
| Adjuvant | 710 | (("Adjuvants, Immunologic/therapeutic use"[Majr]) OR ("Adjuvants, Pharmaceutic/therapeutic use"[Majr])) AND ((english[Filter]) AND (2019:2022[pdat])) |
| Physiologically-based pharmacokinetics | 590 | ((pbpk[Title/Abstract]) OR (Physiologically based[Title/Abstract]) AND ("Pharmacokinetics"[Mesh])) AND ((english[Filter]) AND (2019:2022[pdat])) |
| Adaptive designs | 545 | ((adaptive design*[Title/Abstract]) OR (adaptive trial*[Title/Abstract]) OR ("Adaptive Clinical Trials as Topic"[Mesh])) AND ((english[Filter]) AND (2019:2022[pdat])) |
| Controlled-release technologies | 443 | ("Delayed-Action Preparations/chemistry"[Majr]) AND ((english[Filter]) AND (2019:2022[pdat])) |
| Genetically modified organism(s) | 441 | "Organisms, Genetically Modified"[Majr] NOT "Food"[Mesh] NOT "Plants"[Mesh] AND ((english[Filter]) AND (2019:2022[pdat])) |
| Other in silico models | 326 | ((in silico model*[tiab]) AND ("Computer Simulation"[Mesh]) AND ("Humans"[Mesh])) AND ((english[Filter]) AND (2019:2022[pdat])) |
| Closed loop systems | 307 | (closed loop[Title/Abstract]) AND ("Infusion Pumps"[Mesh]) AND ((english[Filter]) AND (2019:2022[pdat])) |
| Other smart/advanced material | 278 | "Smart Materials"[Majr] AND ("english"[Language] AND 2019/01/01:2022/12/31[Date - Publication]) |
| Other associated medical device | 257 | (("Equipment and Supplies"[Mesh])) AND (("Pharmaceutical Preparations/instrumentation"[Mesh]) OR ("Drug Delivery Systems/instrumentation"[Mesh]) OR ("Drug Development/instrumentation"[Mesh]) OR ("drug development"[Title/Abstract]) OR ("pharmaceutical development"[Title/Abstract])) NOT ("Lab-On-A-Chip Devices"[Mesh]) AND ((english[Filter]) AND (2019:2022[pdat])) |
| Transgenic technologies | 216 | (("Transgenes"[Mesh]) OR (transgen*[Title/Abstract]) OR ("gene transfer"[Title/Abstract])) AND (("Drug Development"[Mesh]) OR (drug development[Title/Abstract]) OR (pharmaceutical development[Title/Abstract])) AND ((english[Filter]) AND (2019:2022[pdat])) |
| Biodefense / biowarfare | 215 | ("Biological Warfare"[Mesh] OR biodefense[tiab] OR biowarfare[tiab]) AND ("english"[Language] AND 2019/01/01:2022/12/31[Date - Publication]) |
| Bayesian designs | 206 | ((bayes*[Title/Abstract]) OR ("Bayes Theorem"[Mesh])) AND ((trial design[tiab]) OR ("Clinical Trials as Topic/methods"[Mesh])) AND ((english[Filter]) AND (2019:2022[pdat])) |
| Novel endpoints | 133 | ((novel endpoint*[Title]) OR (novel end point*[Title]) OR (surrogate endpoint*[Title]) OR (surrogate end point*[Title])) AND ((english[Filter]) AND (2019:2022[pdat])) |
| Platform/Umbrella/basket trials | 97 | (((platform trial*[Title/Abstract]) OR (umbrella trial*[Title/Abstract]) OR (basket trial*[Title/Abstract])) AND ("Clinical Trials as Topic"[Mesh])) AND ((english[Filter]) AND (2019:2022[pdat])) |
| Medicines for tropical diseases | 90 | ((tropical[Title/Abstract]) AND (("Neglected Diseases/drug therapy"[Mesh]) OR ("Tropical Medicine/drug therapy"[Mesh]))) AND ((english[Filter]) AND (2019:2022[pdat])) |
| Extrapolation proposed | 87 | ("Clinical Trials as Topic"[Mesh]) AND (extrapolation[tiab]) AND ((english[Filter]) AND (2019:2022[pdat])) |
| Avatar, nude and humanised mice | 87 | ((nude[Title/Abstract]) OR (avatar[Title/Abstract]) OR (humani*[Title/Abstract])) AND (("Mice"[Mesh]) AND ("Disease Models, Animal"[Mesh])) AND ((english[Filter]) AND (2019:2022[pdat])) AND (review[Filter] OR systematicreview[Filter]) |
| Human cell based in vitro models, Human stem cell in vitro models | 51 | ((in vitro cell based model*[Title/Abstract]) OR (cell based in vitro model*[Title/Abstract])) AND ((english[Filter]) AND (2019:2022[pdat])) |
| New/uncommon pharm. form or route of admin. | 48 | (("Dosage Forms/trends"[Mesh]) OR ("Drug Administration Routes/trends"[Mesh])) AND ((english[Filter]) AND (2019:2022[pdat])) |
| Distributed manufacturing | 43 | (("distributed manufactur*"[Title/Abstract]) OR ("distributed produc*"[Title/Abstract]) OR ("distributed fabric*"[Title/Abstract])) AND ((english[Filter]) AND (2019:2022[pdat])) |
| Bedside/point of care manufacturing | 26 | ((point of care manufactur*[Title/Abstract]) OR (point of care produc*[Title/Abstract]) OR (POC manufactur*[Title/Abstract]) OR (POC produc*[Title/Abstract]) OR ("bedside manufactur*"[Title/Abstract]) OR ("bedside produc*"[Title/Abstract])) AND ((english[Filter]) AND (2019:2022[pdat])) |
| Pharmacological chaperone | 24 | Pharmacological chaperone*[tiab] AND "Molecular Chaperones"[Mesh] AND ((english[Filter]) AND (2019:2022[pdat])) |
| Novel/uncommon excipient | 22 | ("Excipients"[Mesh]) AND (novel excipient*[tiab] OR uncommon excipient*[tiab] OR new excipient*[tiab]) AND ((english[Filter]) AND (2019:2022[pdat])) |
| Mobile/portable manufacturing | 0 | ("mobile manufact*"[tiab] OR ("portable manufact*"[tiab]) AND ((english[Filter]) AND (2019:2022[pdat])) |

Supplementary Table 4. Clinical trials search terms

|  | **Search hits** | **Search terms** |
| --- | --- | --- |
| Other associated medical device | 2994 | Intervention/treatment(device AND drug) \| Phase Early Phase 1, 1, 2, 3 |
| Matrixes | 831 | Other terms(matrix NOT extracellular) \| Phase Early Phase 1, 1, 2, 3 |
| Adjuvant | 813 | Other terms(adjuvant NOT chemotherapy NOT therapy)\| Phase Early Phase 1, 1, 2, 3 |
| Nanotechnologies | 533 | Other terms(nanotechnology OR nano OR nanoformulation OR nanoparticle OR nanovesicle) \| Phase Early Phase 1, 1, 2, 3 |
| Photodynamic product | 355 | Other terms(photodynamic)\| Phase Early Phase 1, 1, 2, 3 |
| Controlled-release technologies | 223 | Other terms(controlled-release) \| Phase Early Phase 1, 1, 2, 3 |
| Adaptive designs | 200 | Other terms(adaptive-design OR adaptive-trial) \| Phase Early Phase 1, 1, 2, 3 |
| E/m-health | 171 | Other terms(e-health OR m-health) \| Phase Early Phase 1, 1, 2, 3 |
| Monitoring devices/sensors/systems | 168 | Intervention/treatment(monitor AND device) \| Phase Early Phase 1, 1, 2, 3 |
| Real world data analysis | 133 | Other terms(real-world OR RWE OR RWD)\| Phase Early Phase 1, 1, 2, 3 |
| Platform/Umbrella/basket trials | 126 | Other terms(platform-trial OR platform-design OR umbrella-trial OR umbrella-design OR basket-trial OR basket-design) \| Phase Early Phase 1, 1, 2, 3 |
| Closed loop systems | 117 | Other terms(closed-loop) \| Phase Early Phase 1, 1, 2, 3 |
| Novel biomarkers, omics | 72 | Other terms(novel-biomarker OR new-biomarker OR omics) \| Phase Early Phase 1, 1, 2, 3 |
| Biomaterials | 53 | Other terms(biomaterial) \| Phase Early Phase 1, 1, 2, 3 |
| New/uncommon pharm. form or route of admin. | 53 | Other terms((new-form AND pharmaceutical) OR (novel-form AND pharmaceutical) OR new-route-of-administration OR novel-route-of-administration OR new-administration-route OR novel-administration-route OR new-delivery-route OR novel-delivery-route) \| Phase Early Phase 1, 1, 2, 3 |
| Transgenic technologies | 53 | Intervention/treatment(transgene OR transgenic) \| Phase Early Phase 1, 1, 2, 3 |
| Extrapolation proposed | 42 | Other terms((extrapolation OR extrapolate) NOT (infinity OR infinite OR AUC0 OR "AUC(0"))\| Phase Early Phase 1, 1, 2, 3 |
| Medicines for tropical diseases | 34 | Condition or disease(tropical) \| Phase Early Phase 1, 1, 2, 3 |
| Organoids | 20 | Other terms(organoid)\| Phase Early Phase 1, 1, 2, 3 |
| Bayesian designs | 16 | Other terms(bayesian-design OR bayesian-trial) \| Phase Early Phase 1, 1, 2, 3 |
| Targeted release to specific site(s) | 16 | Other terms(targeted-release OR targeted-delivery) \| Phase Early Phase 1, 1, 2, 3 |
| Avatar, nude and humanised mice | 12 | Other terms(avatar-mouse OR avatar-mice OR humanised-mouse OR humanised-mice OR humanized-mouse OR humanized-mice OR nude-mouse OR nude-mice)\| Phase Early Phase 1, 1, 2, 3 |
| 3D printing | 9 | Other terms(3d printing OR 3d printed OR 3D printer) \| Phase Early Phase 1, 1, 2, 3 |
| Genome editing (all subtopics) | 8 | Other terms(genome-editing OR genome-engineering) \| Phase Early Phase 1, 1, 2, 3 |
| Physiologically-based pharmacokinetics | 8 | Other terms(Physiologically-based-pharmacokinetic OR Physiological-based-pharmacokinetic OR Physiologically-based-pharmacokinetics OR Physiologically-based-PK OR PB-pharmacokinetic OR PB-pharmacokinetics OR PB-PK OR PBPK)\| Phase Early Phase 1, 1, 2, 3 |
| Novel endpoints | 7 | Other terms(novel-endpoint OR new-endpoint) \| Phase Early Phase 1, 1, 2, 3 |
| Biodefense/biowarfare | 4 | Other terms(biodefense OR biowarfare) \| Phase Early Phase 1, 1, 2, 3 |
| Novel/uncommon excipient | 4 | Other terms(novel-excipient OR new-excipient OR uncommon-excipient)\| Phase Early Phase 1, 1, 2, 3 |
| Synthetic biology | 4 | Other terms( synthetic-biology OR synthetic-biological OR synthetic-gene OR synthetic-genetics OR synthetic-genomics OR gene-synthesis) \| Phase Early Phase 1, 1, 2, 3 |
| Other in silico models | 3 | Other terms(in-silico model)\| Phase Early Phase 1, 1, 2, 3 |
| Big data analysis | 2 | Other terms(big-data OR big-data-analysis)\| Phase Early Phase 1, 1, 2, 3 |
| Genetically modified organism(s) | 2 | Other terms(genetically-modified organism) \| Phase Early Phase 1, 1, 2, 3 |
| Pharmacological chaperone | 2 | Other terms(Pharmacological-chaperone)\| Phase Early Phase 1, 1, 2, 3 |
| Human cell based in vitro models, Human stem cell in vitro models | 1 | Other terms(cell-based in-vitro model) \| Phase Early Phase 1, 1, 2, 3 |
| Other smart/advanced material | 1 | Other terms(smart-material OR advanced-material)\| Phase Early Phase 1, 1, 2, 3 |
| Bedside/point of care manufacturing | 0 | Other terms("bedside manufacturing" OR "point of care manufacturing" OR "POC manufacturing" OR "bedside production" OR "point of care production" OR "POC production") \| Phase Early Phase 1, 1, 2, 3 |
| Bioenhancer | 0 | Other terms(bioenhancer)\| Phase Early Phase 1, 1, 2, 3 |
| Distributed manufacturing | 0 | Other terms("distributed manufacturing") \| Phase Early Phase 1, 1, 2, 3 |
| Mobile/portable manufacturing | 0 | Other terms("mobile manufacturing" OR "portable manufacturing") \| Phase Early Phase 1, 1, 2, 3 |

Supplementary Table 5. Grouping of enabling technologies

| Advanced manufacturing | Mobile/portable manufacturing |
| --- | --- |
|  | Bedside/point of care manufacturing |
|  | Distributed manufacturing |
|  | 3D printing |
|  | Printing |
| Associated medical devices | Other associated medical device |
|  | Matrixes |
| Biomaterials | Biomaterials |
| Delivery methods | New/uncommon pharm. form or route of administration |
|  | Controlled-release technologies |
|  | Targeted release to specific site(s) |
| Development-related: clinical | Medicines for tropical diseases |
|  | Biodefense/biowarfare |
| Digital healthcare | Closed loop systems |
|  | Monitoring devices/sensors/systems |
|  | E/m-health |
| Genetically modified organism(s) | Genetically modified organism(s) |
| Genome editing | Genome editing (all subcategories) |
| Human cell-based in vitro models | Human cell based in vitro models, Human stem cell in vitro models |
| Nanotechnologies | Nanotechnologies |
| Novel biomarkers, omics | Novel biomarkers, omics |
| Novel clinical trial methodologies | Extrapolation proposed |
|  | Platform/Umbrella/basket trials |
|  | Novel endpoints |
|  | Bayesian designs |
|  | Adaptive designs |
| Novel data sources | Big data analysis |
|  | Real world data analysis |
| Novel non-clinical development methods | Avatar, nude and humanised mice |
|  | Other in silico models |
|  | Physiologically-based pharmacokinetics |
|  | Organoids |
| Other ingedients | Novel/uncommon excipient |
|  | Pharmacological chaperone |
|  | Adjuvant |
|  | Bioenhancer |
| Other innovation aspect / enabling technology | Other innovation aspect / enabling technology |
| Smart materials and active substance(s) | Other smart/advanced material |
|  | Photodynamic product |
| Synthetic biology | Synthetic biology |
| Transgenic technologies | Transgenic technologies |
